# Empathy and embodied self: Neural underpinnings of interpersonal relations

**DOI:** 10.1192/j.eurpsy.2021.33

**Published:** 2021-08-13

**Authors:** V. Gallese

**Affiliations:** Medicine and Surgery – Unit of Neuroscience, University of Parma, Parma, Italy

## Abstract

In spite of the historically consolidated psychopathological perspective, neuroscientific research applied to schizophrenia has so far almost entirely neglected the first-person experiential dimension of this syndrome, mainly focusing on higher-order cognitive functions such as executive function, working memory, theory of mind, and the like. An alternative view posits that schizophrenia is a self-disorder characterized by anomalous self-experience and awareness. This view may not only shed new light on the psychopathological features of psychosis but also inspire empirical research targeting the bodily and neurobiological changes underpinning this disorder. Recent empirical evidence on the neurobiological basis of a minimal notion of the self, the bodily self, will be presented. The relationship between the body, its motor potentialities and the notion of minimal self will be illustrated. I will posit that this approach can shed new light on the self-disturbances and social deficits characterizing schizophrenia. I will propose that cognitive neuroscience can today address classic topics of psychopathology by adding a new level of description, finally enabling the correlation between the first-person experiential aspects of psychiatric diseases and their neurobiological roots. To this purpose, I’ll describe putative neural mechanisms underpinning the blurring of self-other distinction in schizophrenic patients. I will posit that brain function anomalies of multisensory integration, differential processing of self- and other-related bodily information, mediating self-experience, might be at the basis of the imbalance in the pre-reflective relationship of the embodied self to the social world observed in schizophrenia.

**Disclosure:**

No significant relationships.

